# Assessment of Ab Initio and Density Functional Theory Methods for the Excitations of Donor-Acceptor Complexes: The Case of the Benzene-Tetracyanoethylene Model

**DOI:** 10.3390/ijms19041134

**Published:** 2018-04-10

**Authors:** Peng Xu, Cai-Rong Zhang, Wei Wang, Ji-Jun Gong, Zi-Jiang Liu, Hong-Shan Chen

**Affiliations:** 1 State Key Laboratory of Advanced Processing and Recycling of Non-ferrous Metals, Lanzhou University of Technology, Lanzhou 730050, Gansu, China; gary316@yeah.net; 2 Department of Applied Physics, Lanzhou University of Technology, Lanzhou 730050, Gansu, China; wangwei3057@163.com (W.W.); jijungong@gmail.com (J.-J.G.); 3 College of Physics and Electronic Engineering, Northwest Normal University, Lanzhou 730070, Gansu, China; chenhs@nwnu.edu.cn; 4 Department of Physics, Lanzhou City University, Lanzhou 730070, Gansu, China; liuzj_scu@126.com

**Keywords:** excited states, ab initio, density functional theory, donor-acceptor complexes, charge transfer

## Abstract

The understanding of the excited-state properties of electron donors, acceptors and their interfaces in organic optoelectronic devices is a fundamental issue for their performance optimization. In order to obtain a balanced description of the different excitation types for electron-donor-acceptor systems, including the singlet charge transfer (CT), local excitations, and triplet excited states, several ab initio and density functional theory (DFT) methods for excited-state calculations were evaluated based upon the selected model system of benzene-tetracyanoethylene (B-TCNE) complexes. On the basis of benchmark calculations of the equation-of-motion coupled-cluster with single and double excitations method, the arithmetic mean of the absolute errors and standard errors of the electronic excitation energies for the different computational methods suggest that the M11 functional in DFT is superior to the other tested DFT functionals, and time-dependent DFT (TDDFT) with the Tamm–Dancoff approximation improves the accuracy of the calculated excitation energies relative to that of the full TDDFT. The performance of the M11 functional underlines the importance of kinetic energy density, spin-density gradient, and range separation in the development of novel DFT functionals. According to the TDDFT results, the performances of the different TDDFT methods on the CT properties of the B-TCNE complexes were also analyzed.

## 1. Introduction

In terms of device architecture and materials [1], promising organic devices (organic photovoltaics, organic light-emitting diodes, organic photon detectors, etc.) usually contain electron donor and acceptor materials constructing an active layer [2,3]. Charge transports in donor/acceptor materials and charge transfer (CT) at the interfaces between electron donors and acceptors have great importance in the improvement of device performance [4,5,6,7,8,9,10,11,12]. Usually, the charge transfer in organic optoelectronic devices involves molecular excitation behavior [13,14]. Molecular excitation properties, such as energy level landscape and local and CT transition characters, determine various photophysical and photochemical properties—including florescence and phosphorescence radiation, internal conversion, intersystem crossing, and other radiationless de-excitation processes—and further affect the electron/hole processes in the device. Therefore, the understanding of the excitation properties of electron donors and acceptors, and in particular of their interfaces, is fundamental to investigating their working principles and to optimizing device performance.

There are several types of ab initio and density functional theory (DFT) methods that can be applied to calculate the electronic excited states of molecular systems. The first type are wavefunction-based methods, including the time-dependent Hartree–Fock (TDHF) theory, the complete active space self-consistent field (CASSCF) method [15], the symmetry-adapted cluster-configuration interaction (SAC-CI) method [16], the equation-of-motion coupled-cluster with single and double excitations (EOM-CCSD) method [17], the configuration interaction singles (CIS) method or the configuration interaction with singles and doubles (CISD) method [18], and other multireference configuration interaction (MRCI) methods. Due to the absence of electronic correlation interaction, TDHF always overestimates the excitation energies. On the other hand, huge computational costs limit the applications of other wavefunction-based methods for large systems even though reasonable accuracy can be achieved by these methods. The second type of method is the many-body theory, also known as the Green function, which is based upon the GW approximation (where G is the Green function and W is the screened Coulomb potential) and the Bethe–Salpeter equation (BSE, altogether the GW-BSE method). The GW-BSE method provides an alternate method for calculating one- and two-particle excitations [19]. In practice, the GW-BSE method usually generates reliable results, but the computational cost for large systems is also too expensive for many important real applications [20], such as the systems related to organic photovoltaics. The third type of method is time-dependent DFT (TDDFT) [21,22]. The moderate computational cost of TDDFT allows it to be performed on large systems [23].

Practical TDDFT calculations are usually performed based upon the adiabatic approximation, which assumes that the exchange-correlation potential at each moment in time depends only on the contemporaneous density [24]. Actually, the functionals in DFT and the methods for solving the TDDFT equations affect the computational accuracy. The most practical applications of the full TDDFT equations are calculated by using linear response theory in which the molecular system is affected by a time-periodic weak perturbation [20]. Through the frequency variation of the effective perturbation, the dynamic polarizability of the molecular system can be calculated, resulting in electronic excitation energies and oscillator strengths [25]. An alternative method for solving TDDFT equations is the Tamm–Dancoff approximation (TDA, altogether TDA-TDDFT) [26], which is a TDDFT analogue of the CIS method [27]. Unlike the full TDDFT, where both excitations and de-excitation matrix elements are considered, the de-excitation contributions in the TDA are neglected because of the weak coupling of matrix elements between the excitations and the de-excitation contributions [28]. It was reported that the application of the TDA significantly improves the description of molecular singlet and triplet excitations [29], such as the correct state ordering in naphthalene and the triplet instability problem [28].

Common practical approximate functionals in DFT include local-density approximation, generalized gradient approximation (GGA), and the conventional hybrid functionals, which employ non-local exchange-correlation potential operators [30]. However, the conventional hybrid functionals, such as B3LYP [31,32,33] and PBE0 [34], usually underestimate the CT excitation energy due to their incorrect long-range asymptotic behavior of exchange-correlation potential [30]. To remedy this deficiency, various functionals, including meta-GGA-based functionals and long-range corrected range-separated hybrid (LRCRSH) functionals, have been developed. The meta-GGA functionals, such as the M06-series functionals [35], contain kinetic energy spin densities, spin-density gradients with several fitted empirical parameters, and variations of exact Hartree–Fock (HF) exchange. These types of functionals provide a good description of CT, non-covalent interaction, main-group thermochemistry and kinetics, etc. For LRCRSH functionals, the Coulomb potential operator is divided into short-range (SR) and long-range (LR) parts by using error function

(1) $$\frac{1}{r}=\frac{erf(\omega r)}{r}+\frac{erfc(\omega r)}{r}$$

where ω is the range-separation parameter [36]. Correspondingly, the SR exchange is usually represented by local or semi-local potential, whereas the LR exchange is adopted as exact HF exchange. The ω is determined semiempirically. For instance, the ω for the LC-ωPBE functional is 0.4 a_0_^−1^ [37]. It should be mentioned that the dynamic exchange-correlation kernel was presented for effective SR electron-electron interaction, and its accuracy can compete with the actual static exchange-correlation kernels [38,39]. Due to the correct LR asymptotic behavior, LRCRSH functionals generate more accurate results for LR CT. Recently, it was reported that the ω can be tuned based upon Koopmans’ theorem to obtain certain expected physical quantities, such as ionization potential and fundamental gap [40,41,42,43]. Also, it was pointed out that the optimally tuned ω depends on the system under study [44]. It is therefore more accurate to say that the optimally tuned ω depends not only on the system and state, but also on the properties (CT or others). The LRCRSH functionals with the optimally tuned ω can generate sufficiently accurate results for many systems [45,46,47,48,49]. But the important issue that should be noted is that if we adopt the LRCRSH functionals with the optimally tuned *ω* for different systems, the calculated results will be rooted in different exchange potentials in DFT. Undoubtedly, this reduces the comparability of computational studies for different systems, which is important in the design of novel materials.

The performance of some DFT functionals in CT of donor/acceptor complexes, such as tetrathiafulvalene-tetracyanoquinodimethane (TTF-TCNQ) [50,51,52] and pentacene-C60 [45,53,54], have been evaluated. Several DFT functionals, including M11 [55], SOGGA11X [56], N12SX/MN12SX [57], HISSbPBE [58], and APF [59] had been presented and implemented in a quantum chemistry package recently. But their performances in molecular singlet/triplet excitations (CT or local excitations) were not systematically evaluated. Meanwhile, regarding the full TDDFT and TDA-TDDFT, which one is the better choice for the description of singlet and triplet excitations for molecular systems? Furthermore, in order to generate a balanced description of singlet/triplet CT and local excitations, is it possible to find a general method without further tuning the empirical parameters (such as the optimally tuned range-separation parameter ω) and do so at an affordable computational cost? Therefore, in this work, in order to highlight the effects of the theoretical methods and to avoid experimental uncertainties (temperature, vibronic coupling, and zero-point effects etc. [60]), the selected benchmark is the EOM-CCSD method because of its higher accuracy for molecular excitation [61,62]. The robust computation of excitation energies by the EOM-CCSD method has been further approved in [63]. Based on the benchmark calculations, we assessed several wavefunction-based methods and the TDDFT methods (both the full TDDFT and the TDA-TDDFT) with different functionals for the excitations of the benzene-tetracyanoethylene (B-TCNE) complexes, which was selected as the reference donor-acceptor model system.

## 2. Results and Discussion

### 2.1. The Exchange-Correlation Energy in the Tested DFT Functionals

In order to understand the different performances of the excited-state calculations, it is necessary to analyze the exchange-correlation energy functionals. The tested DFT functionals in this work can be classified into four types. The first is global hybrid functionals, including the SOGGA11X and APF functionals. The second type of functional is range-separated hybrid functionals, which include LC-ωPBE, ωB97XD, N12SX/MN12SX, and HISSbPBE. Hybrid meta-GGA M06-2X [64] is the third type of functional. The fourth type of functional is the range-separated hybrid meta-GGA functional M11. In order to clearly present the differences, the exchange-correlation energy functionals are listed in Table 1, where the superscripts and subscripts in the formulas stand for method and exchange-correlation energy terms, respectively. For instance, the $E_{X}^{HF}$, $E_{X}^{LR-HF}$, $E_{X}^{SR-M11}$ and $E_{C}^{M11}$ in the expression for the M11 functional are HF exchange, LR HF exchange, SR M11 exchange, and M11 correlation, respectively. During the development of the DFT functionals, because the entire exchange–correlation hole obeys the sum rule (the exchange-correlation hole density integrates to one) and the correlation/exchange hole density must integrate to zero/one, the developed DFT functionals paid more attention to exchange, particularly to the content of exact HF exchange.

### 2.2. Singlet Excitations

**Figure 1:**
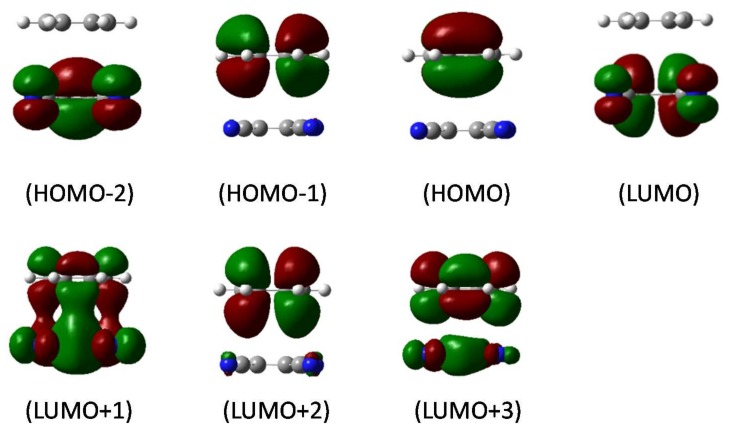
The frontier molecular orbitals involved in charge transfer and local transitions of the benzene-tetracyanoethylene (B-TCNE) complexes via the equation-of-motion coupled-cluster with single and double excitations (EOM-CCSD/SVP) method. The contour threshold of 0.002 a.u. has been applied.

**Figure 2:**
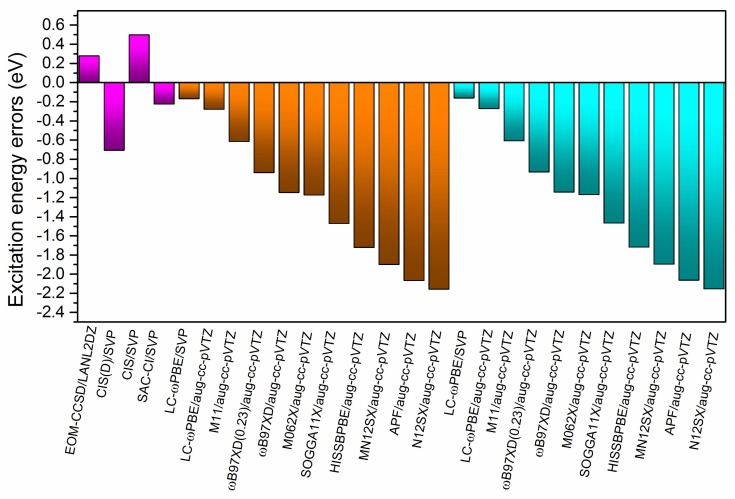
The calculated excitation energy errors relative to the EOM-CCSD/SVP benchmark result for the lowest singlet charge transfer excited state. The purple, brown and blue regions represent the results of ab initio, full time-dependent DFT (TDDFT) and Tamm–Dancoff approximation (TDA)-TDDFT methods, respectively.

**Figure 3:**
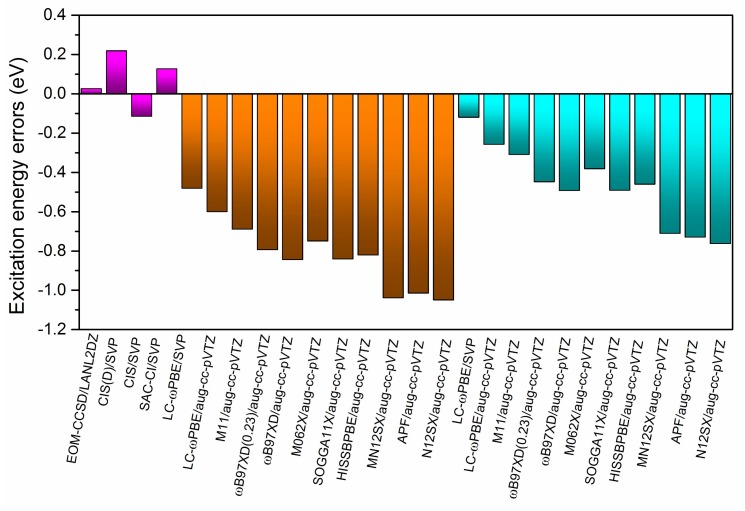
The calculated excitation energy errors relative to the EOM-CCSD/SVP benchmark result for the lowest singlet local excited state. The purple, brown, and blue regions represent the results of the ab initio, full TDDFT, and TDA-TDDFT methods, respectively.

The EOM-CCSD/SVP benchmark results for the B-TCNE complexes are listed in Table 2, including three singlet and triplet excitation energies (eV), wavelength (nm), and main transition configurations with coefficients larger than 10%. Figure 1 shows the molecular orbitals (MOs) involved in these transitions. In terms of the MOs in Figure 1 and transition configurations in Table 2, the first singlet excited state for the B-TCNE complexes can be assigned as the CT excitation (labeled as S-CT_1_), while the main character of the third singlet excited state can be assigned as the locally excited (LE) state (labeled as S-LE_1_), though it exhibits hybridization of the LE state and CT. The calculated vertical electronic excitation energies (eV) of the S-CT_1_ and S-LE_1_ for the B-TCNE complexes are listed in Table 3. The EOM-CCSD/SVP benchmark results of the S-CT_1_ and S-LE_1_ are 4.19 and 5.34 eV, respectively. In order to give the computational methods accuracy, the calculated excitation energy errors (defined as the excitation energy difference between the giving method and the benchmark) of S-CT_1_ and S-LE_1_ are presented in Figure 2 and Figure 3.

The excitation energies of S-CT_1_ and S-LE_1_ calculated with EOM-CCSD/LanL2DZ are about 0.28 and 0.03 eV higher than those of the EOM-CCSD/SVP results, underlining that the basis sets incompleteness generate more significant effects on the CT excitations than those on the LE state. The excitation energies of S-CT_1_ and S-LE_1_ calculated by LC-ωPBE/SVP are about 0.11 eV higher than those of LC-ωPBE/aug-cc-pVTZ. The tendencies indicated by these data are that (i) the larger basis sets generate smaller singlet CT and LE energies and (ii) the dependence of ab initio methods are more sensitive on basis sets than those of DFT methods. The TDA results of LC-ωPBE/aug-cc-pVTZ and LC-ωPBE/SVP also support these tendencies.

For the results of the wavefunction-based methods, the error of S-CT_1_ excitation energies of the CIS and CIS(D) methods are about 0.50, −0.71 eV relative to the benchmark results, and the errors of S-LE_1_ are about −0.12, 0.22 eV for the CIS and CIS(D) methods, respectively. The errors of the CIS and CIS(D) methods for S-CT_1_ and S-LE_1_ indicate that increasing configurations cannot ensure the improvement of accuracy because of insufficient electronic correlation. The SAC-CI method results for S-CT_1_/S-LE_1_ excitation energies are about 0.23/0.13 eV lower/higher than those of the benchmark, indicating the accuracy improvement of the CT excitation relative to those of the CIS and CIS(D) methods by configuration selection.

For the full-TDDFT results with aug-cc-pVTZ basis sets, the excitation energies of S-CT_1_ and S-LE_1_ range from 2.03~3.91 eV and 4.29~4.74 eV, respectively. For the global hybrid functionals, the S-CT_1_ and S-LE_1_ excitation energies of SOGGA11X/APF are about 1.47/2.07 and 0.84/1.01 eV lower than those of benchmark results, respectively. For the range-separated functionals, the S-CT_1_ and S-LE_1_ excitation energies of LC-ωPBE/ωB97XD/ωB97XD(0.23)/N12SX/MN12SX/HISSbPBE are about 0.28/1.15/0.94/2.16/1.90/1.72 and 0.60/0.84/0.79/1.05/1.04/0.82 eV lower than the benchmark results, respectively. The good performance of the LC-ωPBE functional can be attributed to its range-separation parameter, which is relatively suitable for the B-TCNE complexes. The ωB97XD and ωB97XD(0.23) results indicate that the LRCRSH functionals with the optimally tuned range-separation parameter ω improve computational accuracy for singlet CT and LE excitations through the variation of HF exchange in $E_{XC}$ For the hybrid meta-GGA functional, the S-CT_1_ and S-LE_1_ excitation energies of M06-2X are about 1.18 and 0.75 eV lower than those of benchmark results, respectively. The accuracy of the M06-2X functional for singlet excitations is similar to those of the ωB97XD and ωB97XD(0.23) functionals due to its high nonlocality functional with double the amount of non-local exchange [35]. For the range-separated hybrid meta-GGA M11 functional, the S-CT_1_ and S-LE_1_ excitation energies are about 0.62 and 0.69 eV lower than those of benchmark results, respectively, generating a balanced description of the LE state and the CT excitation with fewer excitation energy errors. According to these descriptions and the data presented in Figure 2 and Figure 3, for most of the tested DFT functionals, the excitation energy errors are not systematic, and the CT excitation energy errors are larger than those of the LE state because the delocalization errors in $E_{XC}$ generate more significant influence on the CT excitations. The exchange-correlation kernel in the tested DFT functionals contains local/semi-local potential and non-local HF exchange to different extents, meaning that the delocalization errors cannot be completely dismissed and can be partially remedied by increasing HF exchange in the DFT functionals. Sini et al. reported that, for TTF-TCNQ complexes, the more content of HF exchange in the DFT functionals, the higher the CT excitation energy [65]. In this case, the smaller content of HF exchange in the DFT functionals, such as the APF/MN12SX/N12SX/HISSbPBE functionals, the larger the excitation energy errors (for both the CT and the LE state). 

For the TDA-TDDFT results with aug-cc-pVTZ basis sets (see Table 3), the excitation energies of S-CT_1_ and S-LE_1_ range from 2.03~3.91 eV and 4.58~5.08 eV, respectively. The excitation energy errors of S-CT_1_ and S-LE_1_, which are presented in Figure 2 and Figure 3, range from 0.27~2.16 eV and 0.26~0.76 eV, respectively. The functional effects in the TDA-TDDFT are similar to those in the full TDDFT. Compared with the results of the full TDDFT, the TDA-TDDFT significantly improves the accuracy of LE (about 0.3 eV), underlining the rationality of ignoring the de-excitation contributions in the TDA-TDDFT. But the accuracy improvement of the CT is tiny, suggesting that the de-excitation contributions for the LE state are more remarkable than those for CT. Another possible reason for the improvement of the TDA-TDDFT might be the compensation effects between the exchange-correlation DFT kernel and the TDA. 

### 2.3. Triplet Excitations

**Figure 4:**
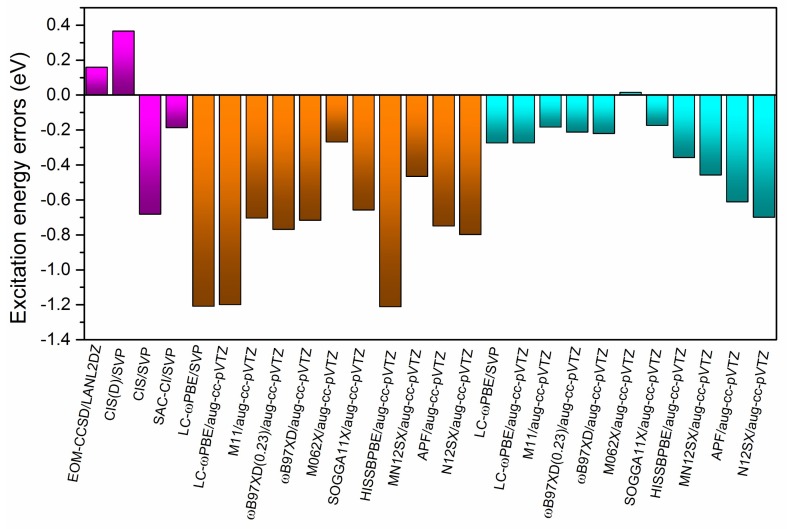
The calculated excitation energy errors relative to the EOM-CCSD/SVP benchmark result for the lowest triplet excited state. The purple, brown, and blue regions represent the results of the ab initio, full TDDFT, and TDA-TDDFT methods, respectively.

In terms of triplet excitations data (listed in Table 2) and related MOs presented in Figure 1, the first triplet excited state (labeled as T_1_) is LE in TCNE with π→π* transition, and the EOM-CCSD/SVP benchmark excitation energy is 2.71 eV. The T_1_ excitation energy of the B-TCNE complexes calculated by the selected ab initio and DFT methods are also listed in Table 3. The calculated excitation energy errors are shown in Figure 4. The T_1_ excitation energy of the SAC-CI result is about 0.19 eV smaller than that of the benchmark. The excitation errors indicate that the SAC-CI method is superior to the CIS and CIS(D) methods for triplet excitations. For the full TDDFT results, the calculated T_1_ excitation energy range from 1.50~2.44 eV, and the corresponding excitation energy errors are about 0.27~1.21 eV. The M06-2X functional generates the smallest excitation error (~0.27 eV). The excitation energy errors of the LC-ωPBE and HISSbPBE functionals are larger than those of other functionals, indicating that these functionals are not suitable for triplet excitations. The other functionals have similar accuracy. The common feature of the LC-ωPBE and HISSbPBE functionals are their PBE correlation. In spin-flip transition, the importance of electronic correlation is increasing because correlation exists in electrons with opposite spin. For the TDA-TDDFT results, the T_1_ excitation energies range from 2.01~2.72 eV, and the accuracy of the T_1_ excitation energies is significantly improved. It was found that applying TDA upshifts the triplet excitation energies and reduces the triplet excitation errors for the selected organic molecules [66]. The T_1_ excitation energy difference between the TDA-TDDFT results and the full TDDFT results also emphasizes the remarkable de-excitation contributions in the full TDDFT and again underlines the rationality of ignoring the de-excitation contributions in the TDA-TDDFT for triplet excitation. Meanwhile, the performances of the M06-2X and M11 functionals in the TDA-TDDFT are superior to that of other functionals, and the excitation energy errors of the APF/MN12SX/N12SX/HISSbPBE functionals, which contain small contents of HF exchange are larger than those of other functionals. In addition, it was reported that the adiabatic approximation error for the S_0_→T_1_ spin-flip transition is larger than that for the singlet transition [67]. Therefore, adiabatic approximation of TDDFT is another reason for excitation energy error.

### 2.4. Assessment of Different Methods for Excited States

In order to quantitatively evaluate the performance of the different computational methods for the excitations of the donor-acceptor B-TCNE complexes, we defined the arithmetic mean of the absolute errors (AMAE) and standard errors (SE) of the vertical electronic excitation energies for the B-TCNE complexes as

(2) $$\mathrm{AMAE}=\frac{\sum_{i}|E_{i}^{m}-E_{i}|}{N}$$

and

(3) $$\mathrm{SE}=\sqrt{\frac{\sum_{i}{\left(E_{i}^{m}-E_{i}\right)}^{2}}{N}}$$

respectively. In these expressions, the *i* presents the lowest singlet local/CT excitation or the first triplet excited state, *N* = 3 means the average of the abovementioned three kinds of excited states, the *E_i_* is the corresponding benchmark excitation energies calculated with EOM-CCSD/SVP, and the $E_{j}^{m}$ is the corresponding excitation energies calculated with other methods. In terms of the EOM-CCSD/SVP benchmark results, Table 4 lists the calculated AMAE and SE of the vertical excitation energies for the B-TCNE complexes. Compared with the CIS and CIS(D) results, the smaller AMAE and SE of the SAC-CI method mean it performs better in excitation calculations. According to the full TDDFT results, the smallest SE of the M11 functional indicates that it performs best in showing a balanced description of singlet LE/CT and triplet excitations for the B-TCNE complexes. For the TDA-TDDFT, the M11 functional has the smallest SE except for the LC-ωPBE functional. The AMAE and SE calculated using LC-ωPBE/aug-cc-pVTZ and (TDA) LC-ωPBE/aug-cc-pVTZ supports the finding that the smallest AMAE and SE of (TDA) LC-ωPBE/aug-cc-pVTZ results from TDA, rather than a DFT functional. Furthermore, the AMAE and SE for a given functional in the TDA-TDDFT are smaller than those in the full TDDFT, underling the effectiveness of TDA in excitation calculations.

It should be noted that the benchmark EOM-CCSD data raises uncertainty because of the relatively small basis sets of SVP. For the B-TCNE complexes in this work, according to the basis sets effects on the EOM-CCSD data for the tested molecules [62,68] and the low-lying excited states that were calculated in this work, the estimated average uncertainty of the EOM-CCSD data for the S-LE_1_, S-CT_1,_ and T_1_ excitations should be less than 0.1 eV if the basis sets can be extended from SVP to the larger. However, this uncertainty of the EOM-CCSD data does not affect the assessment of the DFT functionals because the AMAE for the excitation energies calculated using the TDDFT methods with different functionals are remarkably larger than the uncertainty of the EOM-CCSD benchmark data.

### 2.5. Transferred Charges from TDDFT

**Figure 5:**
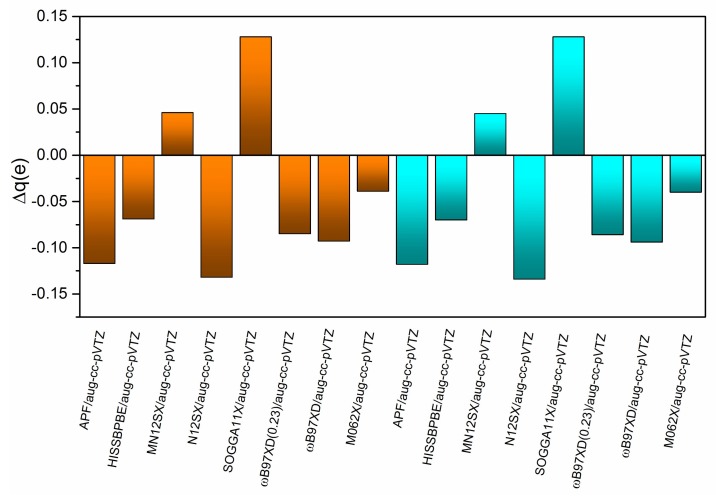
The difference of transferred charges (Δq, in *e*) between the TDDFT results with different functionals and the full TDDFT results with the M11 functional. The brown and blue regions represent the full TDDFT and TDA-TDDFT results, respectively.

In order to further investigate the computational method effects on the CT properties, the calculated transferred charges q_CT_ (in *e*) and the CT excitation length D_CT_ (in Å) for the S-CT_1_ state of the B-TCNE complexes are listed Table 5. Because the electron densities of EOM-CCSD, CIS/CIS(D), SAC-CI, and LC-ωPBE are not available with the density keyword in the Gaussian package, the CT properties were not investigated using these methods. For a given DFT functional, the q_CT_ and D_CT_ results of the full TDDFT are slightly larger than those of the TDA-TDDFT (about 0.001 *e* and 0.001 Å, respectively), suggesting that the full TDDFT and the TDA-TDDFT generate similar electron density variation induced by excitation. The q_CT_ and the CT excitation length D_CT_ range from 1.22 to 1.49 *e*, and from 1.78 to 2.10 Å, respectively. The remarkable q_CT_ confirms the CT character of this excited state. The calculated D_CT_ is smaller than the distance between molecular centers because of polarization effects during CT. It also can be found that the larger q_CT_ corresponds to the shorter D_CT_. For instance, the SOGGA11X functional in the full TDDFT generates the largest q_CT_ (~1.49 *e*), while the D_CT_ is the shortest (~1.78 Å) generated. Considering the accuracy of the M11 functional for excitation energy, the differences of transferred charges (Δq, in *e*) between the TDDFT results with different functionals and the full TDDFT results with the M11 functional are presented in Figure 5 in order to compare the effects of the methods on the CT properties. Apparently, the smallest Δq of the M06-2X functional suggests that the M11 and M06-2X functionals generate similar CT properties for the B-TCNE complexes.

## 3. Computational Method

**Figure 6:**
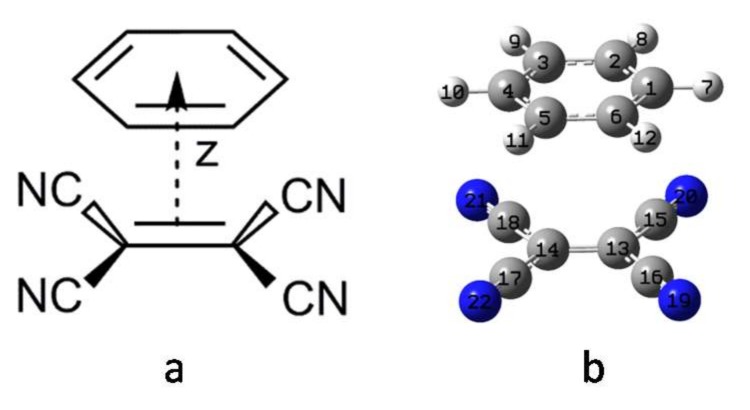
(**a**) The molecular structure of the B-TCNE complex model with cofacial configuration; Z is the distance between molecular centers; (**b**) The cofacial configuration of the B-TCNE complex constructed by the optimized monomer. The atomic serial numbers are also given. The colored circles present different types of atoms (blue: N; grey: C; light grey: H).

The isolated monomers of the benzene and TCNE molecules were fully optimized at the level of the B3LYP functional and aug-cc-pVTZ basis sets [69] with a tight self-consistent field convergence threshold. The optimized benzene and TCNE molecules are planar structures with point groups D_6h_ and D_2h_, respectively. The monomers of benzene and TCNE molecular geometries were frozen to construct the B-TCNE complexes because the intra-molecular monomer geometrical parameters are a little perturbed during the formation of donor-acceptor complexes [44,70]. Hence, the relative orientation of the π-stacked B-TCNE complexes was constructed in parallel cofacial configuration with the fixed distance between the molecular centers along the *z*-axis (see Figure 6, *z*-axis is also labeled). The typical donor-acceptor inter-planar distances were about 3.3–4.0 Å [46,50,51,52,65,71]. In this work, the distance between the benzene and TCNE molecular centers is fixed at 3.5 Å, which is close to the value of the TTF-TCNQ [65] and terthiophene-TCNQ [71] complexes. 

The wavefunction-based methods EOM-CCSD [17], CIS/CIS(D) [18], and SAC-CI [16] were applied to calculate the excited states of the B-TCNE complexes. To compare the effects of the basis sets, the LanL2DZ and SVP were adopted for the EOM-CCSD calculations, while the SVP basis sets were applied in the CIS, CIS(D), and SAC-CI calculations. The EOM-CCSD, CIS, CIS(D), and SAC-CI calculations with aug-cc-pVTZ basis sets are beyond the capability of our computing cluster. In the full TDDFT and TDA-TDDFT calculations, the tested functionals included LC-ωPBE [37], M11 [55], ωB97XD [72], M06-2X [64], SOGGA11X [56], N12SX/MN12SX [57], HISSbPBE [58], and APF [59]. The LC-ωPBE, ωB97XD, and M06-2X functionals were selected for comparison of calculating singlet LE/CT and triplet excitations. The aug-cc-pVTZ basis sets were applied in the TDDFT calculations. To further check the effects of the basis sets, the full TDDFT calculations with the LC-ωPBE functional were also performed at the level of the SVP basis sets. In order to further check the LRCRSH functional performance in the CT and local excitations, the ωB97XD functional with the optimally tuned ω was also tested in the full TDDFT and TDA-TDDFT calculations. The optimally tuned ω value for the B-TCNE complex is 0.23 a_0_^−1^, which was obtained according to the reported tuning procedure [20]. The geometrical optimization and excitation calculations were performed with the Gaussian09 package [73]. The transferred charges were analyzed using the Multiwfn program [74], and the details of the method for analyzing the transferred charges were reported in our previous work [75,76].

## 4. Conclusions

In this work, in order to get a balanced description of different excitation types for electron-donor-acceptor systems, including singlet CT and LE, as well as triplet excited states, B-TCNE complexes were selected as the model system, and then several ab initio methods and the full/TDA-TDDFT were evaluated with different functionals. On the basis of the benchmark calculations of the EOM-CCSD method, the arithmetic mean of the absolute errors and standard errors of the electronic excitation energies for the different computational methods suggest that the accuracy of the SAC-CI method is better than that of the CIS/CIS(D) method, the M11 functional in DFT is superior to the other tested functionals for a balanced description of LE, CT, and triplet excitations, and the TDA-TDDFT improves the accuracy of the calculated excitation energies relative to full TDDFT. The TDA-TDDFT and full TDDFT results give similar transferred charges and CT excitation lengths. Meanwhile, the calculated errors are not systematic and depend on the characters of the excited states. Furthermore, in terms of the exchange-correlation energy formula for the M11 functional, the performance of the M11 functional underlines the importance of kinetic energy density, spin-density gradient, and range separation in the development of novel DFT functionals.

## Figures and Tables

**Table 1:** The exchange-correlation energy of the selected density functional theory (DFT) functionals in this work.

DFT Functionals	$E_{xc}$	Key Parameters
APF	$0.411E_{XC}^{B3PW91}+0.589E_{XC}^{PBE1PBE}$	
SOGGA11X	$\left(\frac{X}{100}\right)E_{X}^{HF}+\left(1-\frac{X}{100}\right)E_{X}^{SOGGA11}+E_{C}^{SOGGA11}$	*x* = 40.15
LC-ωPBE	$E_{X}^{\omega PBE,SR}\left(\omega \right)+E_{X}^{HF,LR}\left(\omega \right)+E_{C}^{PBE}$	ω = 0.4 a_0_^−1^
ωB97XD	$E_{X}^{LR-HF}+C_{X}E_{X}^{SR-HF}+E_{X}^{SR-B97}+E_{C}^{B97}+E_{disp}$	ω = 0.2 a_0_^−1^ *c_x_* = 0.222
N12SX/MN12SX	$\left(\frac{x}{100}\right)E_{X}^{SR-HF}+E_{\mathrm{NXC}}+E_{C}$	*x* = 25 ω = 0.11 a_0_^−1^
HISSbPBE	$E_{X}^{SR-PBE}+\frac{2}{5}E_{X}^{MR-PBE}+\frac{3}{5}E_{X}^{MR-HF}+E_{X}^{LR-PBE}+E_{C}^{PBE}$	$\omega^{SR}$ = 0.84 a_0_^−1^ $\omega^{LR}$ = 0.20 a_0_^−1^
M06-2X	$\left(\frac{X}{100}\right)E_{X}^{HF}+\left(1-\frac{X}{100}\right)E_{X}^{M06-2X}+E_{C}^{M06-2X}$	*x* = 54
M11	$\left(\frac{X}{100}\right)E_{X}^{HF}+\left(1-\frac{X}{100}\right)(E_{X}^{LR-HF}+E_{X}^{SR-M11})+E_{C}^{M11}$	*x* = 42.8 ω = 0.25 a_0_^−1^

**Table 2:** The calculated three singlet and triplet excitation energies (eV), wavelength (nm), and main transition configurations with coefficients larger than 10% for the B-TCNE complexes (EOM-CCSD/SVP).

Excited States	Main Transition Configurations	Excitation Energies (eV/nm)
S1	H→L (90.3%)	4.19/296
S2	H−1→L (90.2%)	4.23/293
S3	H−1→L+2 (42.9%); H→L+3 (31.2%); H→L+1 (13.4%)	5.34/232
T1	H−2→L (86.1%)	2.71/457
T2	H→L+2 (44.8%); H−1→L+3 (32.0%); H−1→L+1 (12.4%)	4.08/304
T3	H→L (89.5%)	4.16/297

**Table 3:** The calculated vertical electronic excitation energies (eV) of the lowest singlet charge transfer (S-CT_1_) and locally excited states (S-LE_1_), as well as the first triplet excited state (T_1_) for the B-TCNE complexes.

Method	S-CT_1_ (eV)	S-LE_1_ (eV)	T_1_ (eV)
EOM-CCSD/LanL2DZ	4.4673	5.3663	2.8692
EOM-CCSD/SVP	4.1882	5.3402	2.7103
CIS(D)/SVP	3.4807	5.5586	3.0764
CIS/SVP	4.6855	5.2257	2.0291
SAC-CI/SVP	3.9641	5.4669	2.5240
LC-ωPBE/SVP	4.0198	4.8595	1.5016
LC-ωPBE/aug-cc-pVTZ	3.9090	4.7404	1.5110
M11/aug-cc-pVTZ	3.5738	4.6509	2.0067
ωB97XD(0.23)/aug-cc-pVTZ	3.2486	4.5466	1.9417
ωB97XD/aug-cc-pVTZ	3.0399	4.4959	1.9934
M062X/aug-cc-pVTZ	3.0142	4.5908	2.4418
SOGGA11X/aug-cc-pVTZ	2.7162	4.4993	2.0527
HISSbPBE/aug-cc-pVTZ	2.4667	4.5193	1.4994
MN12SX/aug-cc-pVTZ	2.2881	4.3012	2.2448
APF/aug-cc-pVTZ	2.1206	4.3254	1.9606
N12SX/aug-cc-pVTZ	2.0305	4.2905	1.9121
(TDA) LC-ωPBE/SVP	4.0263	5.2205	2.4361
(TDA)LC-ωPBE/aug-cc-pVTZ	3.9160	5.0828	2.4365
(TDA)M11/aug-cc-pVTZ	3.5799	5.0313	2.5272
(TDA)ωB97XD(0.23)/aug-cc-pVTZ	3.2547	4.8925	2.4985
(TDA)ωB97XD/aug-cc-pVTZ	3.0455	4.8483	2.4905
(TDA)M062X/aug-cc-pVTZ	3.0191	4.9585	2.7250
(TDA)SOGGA11X/aug-cc-pVTZ	2.7205	4.8491	2.5366
(TDA)HISSbPBE/aug-cc-pVTZ	2.4703	4.8803	2.3527
(TDA)MN12SX/aug-cc-pVTZ	2.2915	4.6294	2.2528
(TDA)APF/aug-cc-pVTZ	2.1242	4.6105	2.0993
(TDA)N12SX/aug-cc-pVTZ	2.0340	4.5779	2.0113

**Table 4:** According to the EOM-CCSD/SVP benchmark results, the listed data are the calculated arithmetic mean of the absolute errors (AMAE) and standard errors (SE) of the vertical electronic excitation energies for the B-TCNE complexes.

Method	AMAE (eV)	SE (eV)
EOM-CCSD/LanL2DZ	0.1547	0.1860
CIS(D)/SVP	0.4306	0.4769
CIS/SVP	0.4310	0.4914
SAC-CI/SVP	0.1790	0.1835
LC-ωPBE/SVP	0.6193	0.7573
LC-ωPBE/aug-cc-pVTZ	0.6928	0.7908
M11/aug-cc-pVTZ	0.6691	0.6702
ωB97XD(0.23)/aug-cc-pVTZ	0.8339	0.8373
ωB97XD/aug-cc-pVTZ	0.9032	0.9211
M062X/aug-cc-pVTZ	0.7306	0.8189
SOGGA11X/aug-cc-pVTZ	0.9902	1.0498
HISSbPBE/aug-cc-pVTZ	1.2511	1.3043
MN12SX/aug-cc-pVTZ	1.1349	1.2789
APF/aug-cc-pVTZ	1.2779	1.3984
N12SX/aug-cc-pVTZ	1.3352	1.4600
(TDA) LC-ωPBE/SVP	0.1853	0.1964
(TDA) LC-ωPBE/aug-cc-pVTZ	0.2678	0.2679
(TDA) M11/aug-cc-pVTZ	0.3668	0.4078
(TDA) ωB97XD(0.23)/aug-cc-pVTZ	0.5310	0.6101
(TDA) ωB97XD/aug-cc-pVTZ	0.6181	0.7294
(TDA) M062X/aug-cc-pVTZ	0.5218	0.7101
(TDA) SOGGA11X/aug-cc-pVTZ	0.7108	0.8992
(TDA) HISSbPBE/aug-cc-pVTZ	0.8451	1.0473
(TDA) MN12SX/aug-cc-pVTZ	1.0217	1.1989
(TDA) APF/aug-cc-pVTZ	1.1349	1.3122
(TDA) N12SX/aug-cc-pVTZ	1.2052	1.3796

**Table 5:** The transferred charges $q_{\mathrm{CT}}$ (in *e*) and the charge transfer excitation length $D_{\mathrm{CT}}$ (in Å) for the first singlet charge transfer excited state of the B-TCNE complexes.

Methods	$q_{CT}$	$D_{CT}$
APF	1.240	2.079
HISSbPBE	1.288	2.045
M11	1.357	2.012
MN12SX	1.403	1.848
N12SX	1.225	2.101
SOGGA11X	1.485	1.784
ωB97XD(0.23)	1.272	2.130
ωB97XD	1.264	2.135
M062X	1.318	2.028
(TDA) APF	1.239	2.078
(TDA) HISSbPBE	1.287	2.045
(TDA) M11	1.357	2.012
(TDA) MN12SX	1.402	1.847
(TDA) N12SX	1.223	2.100
(TDA) SOGGA11X	1.485	1.783
(TDA) ωB97XD(0.23)	1.271	2.130
(TDA)ωB97XD	1.263	2.135
(TDA) M062X	1.317	2.028
